# Supplementary material for: A spatio-temporal individual-based network framework for West Nile virus in the USA: Spreading pattern of West Nile virus
Source: PLoS Comput Biol. 2019 Mar 13;15(3):e1006875. doi: 10.1371/journal.pcbi.1006875 (PMC6433293; doi:10.1371/journal.pcbi.1006875)
Supplement: S1 Text — (PDF) [file pcbi.1006875.s001.pdf]

Spatial distribution of West Nile Virus in 2014 and 2016.

Sifat A. Moon[1], Lee W. Cohnstaedt[2*], D. Scott McVey[2], Caterina M. Scoglio[1]

1 Department of Electrical & Computer Engineering, Kansas State University, Manhattan, Kansas, United States of America
2 Arthropod-Borne Animal Diseases Research Unit, Center for Grain and Animal Health Research, USDA ARS, Manhattan, Kansas, United States of America

* Lee.Cohnstaedt@ARS.USDA.GOV

Supplementary information

In this research, we compared three distance dispersal kernel to understand the spatial distribution of WNV. Distance dispersal kernels are: 1) Exponential kernel, 2) power-law kernel, and 3) power-law influenced by flyway kernel. We used this framework in the USA for 2014-2016. Approximate Bayesian computation based on sequential Monte Carlo sampling (ABC-SMC) was used for parameter estimation and for selection of the best kernel. The results for 2014 and 2016 are given below.

Results for 2014

In 2014, WNV infected human cases in the USA was 2205. All the states were infected except Alaska, North Carolina, and West Virginia. The inputs of this framework for 2014 are weekly human incidence data by states, avian population data by states, and average monthly temperature data by states for 2014. The target and intermediate distributions of model parameter m from ABC-SMC model selection method are given in Fig SA. Bayes factor was calculated from the last population (population-8 in Fig SA). In the last population, exponential kernel was selected for 79 times, general power-law kernel was selected for 96 times, and power-law-flyway was selected for 825 times.

The obtained Bayes factors for 2014 are:

$$B_{3,1} = \frac{825}{79} = 10.4430 \tag{1}$$

$$B_{3,2} = \frac{825}{96} = 8.5938 \tag{2}$$

From the analysis of Bayes factor for 2014, we can conclude that power-law influenced by flyway distance kernel network model has positive evidence against other two kernels. The estimated parameters are provided in table SA.

Performance of the power-law flyway kernel network model for 2014. To see the performance for this framework for 2014, we used estimated parameters from ABC-SMC parameter estimation method for power-law influenced by flyway kernel network model. The parameters are presented in the Table SA. The results from 1000 simulation are aggregated in the box plot of Fig SB. Total observed human cases by states from CDC are given by blue star. We found that from 49 locations, the total human incidence case for 41 locations falls within the range of simulation results. The simulation results could not follow the observed data for Arizona, Colorado, Kansas, Louisiana, Mississippi, Nebraska, New Mexico, and Washington.

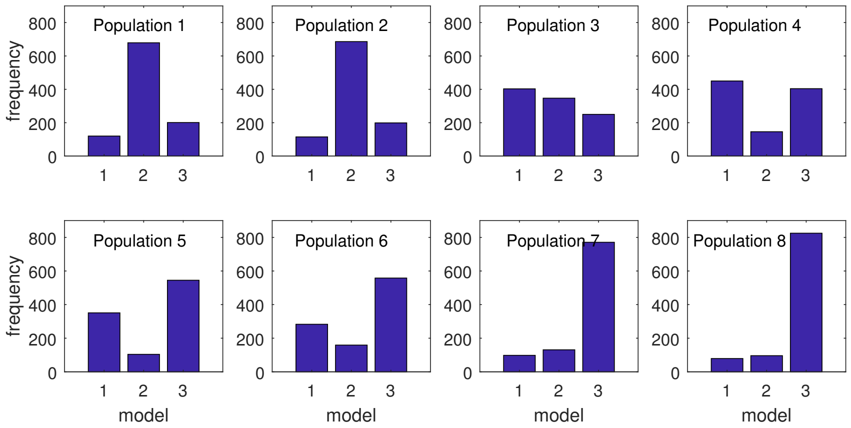

Fig A. Population of the marginal posterior distribution of the three models for 2014. Model-1 represents exponential kernel, model-2 represents power-law kernel, and model-3 represents power-law influenced by flyway kernel. Here, Population-8 is the approximation of the final marginal posterior distribution of model parameter m and population 1-7 are intermediate distributions. Population-0 is the discrete uniform prior distribution, which is not shown here.

Table A. Estimated parameters for power-law biased by flyway network model for 2014, 2015 and 2016 from ABC-SMC parameter estimation algorithm.

Parameter		2014	2015	2016
Network parameter, K	mean	2.4091	2.3147	2.4233
	median	2.3495	2.2690	2.3889
	(95% CI)	(2.3469, 2.4713)	(2.3030, 2.3264)	(2.3353, 2.5114)
Constant for transmission rate, β_0	mean	0.0028 day^{-1}	0.0059 day^{-1}	0.0029 day^{-1}
	median	0.0026 day^{-1}	0.0061 day^{-1}	0.0031 day^{-1}
	(95% CI)	(0.0025, 0.0032 day^{-1})	(0.0058, 0.0059 day^{-1})	(0.0028, 0.0035 day^{-1})
Transition rate from exposed to infectious node, λ	mean	0.0445 day^{-1}	0.0721 day^{-1}	0.0452 day^{-1}
	median	0.0436 day^{-1}	0.0706 day^{-1}	0.0460day^{-1}
	(95% CI)	(0.0434, 0.0455 day^{-1})	(0.0718, 0.0724 day^{-1})	(0.0443, 0.0461 day^{-1})

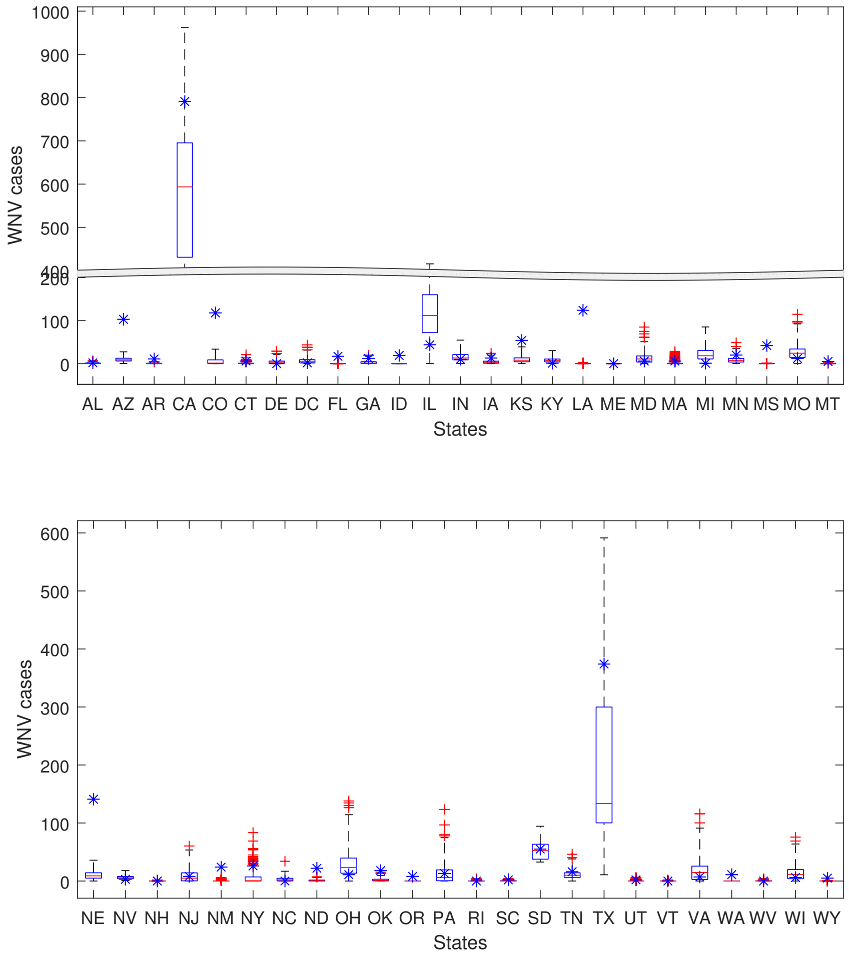

Fig B. WNV human incidence by states for 2014 from power-law influenced by flyway kernel model (for network parameter K=2.4091, constant for transmission rate $\beta_0 = 0.0028 day^{-1}$, and transition rate for exposed to infectious $\lambda = 0.0445 day^{-1}$), generated from 1000 simulation and observed data are indicated by blue colored star points. states name are given in short form. Simulated results are represented with a box plot in which the red horizontal lines show the median and the bottom and top edges of the boxes indicate 25th and 75th percentile respectively, The whiskers show the ranges of data points not considered outliers and outliers are showing by red + symbol. Broken scale is used for sake of visualization.

Results for 2016

In 2016, WNV infected human cases in the USA was 2149. All the states were infected for WNV (except Hawaii and Alaska). 27 states had more than 10 WNV disease cases. California, Colorado, Illinois, South Dakota, and Texas had more than 100 WNV disease cases. This is the most recent year when weekly WNV incidents are publicly available from *CDC* [1]. For host population, we used American Robin population data for 2016 from *eBird* [2]. The description of the host population and sub-networks are provided in Table S3 in the Text S3. We started the epidemic from Arizona because we found highest disease cases in Arizona among all other states before June (in this framework, simulation has started from June). The target and intermediate distributions of model parameter m from ABC-SMC model selection method are shown in Supporting Fig SC. The Bayes factor is obtained from the marginal posterior distribution of m, which we got from the final population (Population 8 in Fig SC). The calculated Bayes factors are:

$$B_{3,1} = \frac{879}{88} = 9.9886 \tag{3}$$

$$B_{3,2} = \frac{879}{33} = 26.6364 \tag{4}$$

From the interpretation of Bayes factors [3], we found positive evidence in favor of power-law influenced by flyway distance kernel network model compared to exponential distance kernel network model and strong evidence in favor of power-law influenced by flyway distance kernel network model compared to power-law distance kernel network model for 2016.

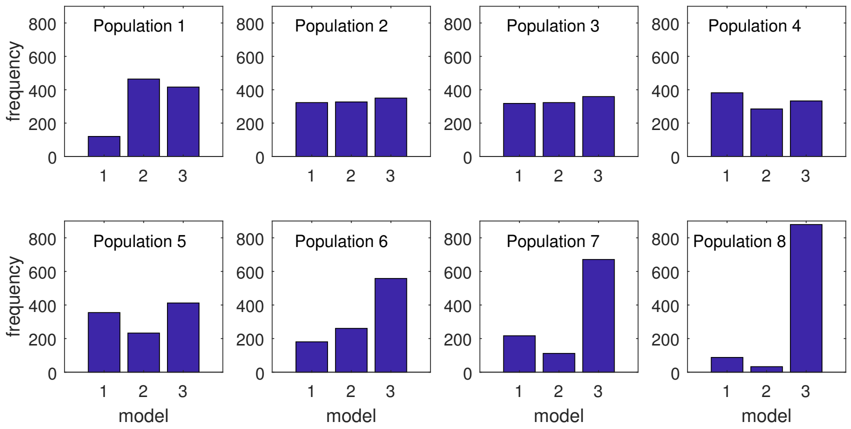

Fig C. Population of the marginal posterior distribution of the three models for 2016. Model-1 represents exponential kernel, model-2 represents power-law kernel, and model-3 represents power-law influenced by flyway kernel. Here, Population-8 is the approximation of the final marginal posterior distribution of model parameter m and population 1-7 are intermediate distributions. Population-0 is the discrete uniform prior distribution, which is not shown here.

Performance of power-law flyway kernel network model for 2016. Fig SD are showing the simulation results of 1000 realizations of the framework for 2016 for power-law-flyway network. We found that, observed data for 45 states among 49 locations falls within the range of the simulated results for 2016. The simulated results could not follow for observed human WNV incidence for Colorado, Louisiana, Mississippi, and North Dakota.

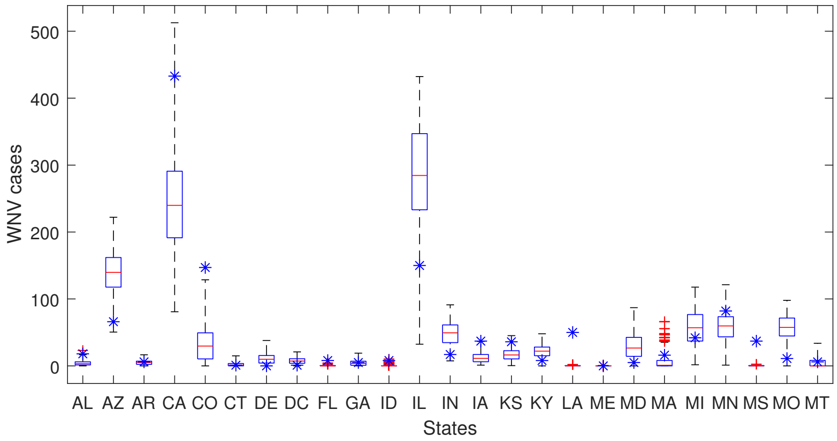

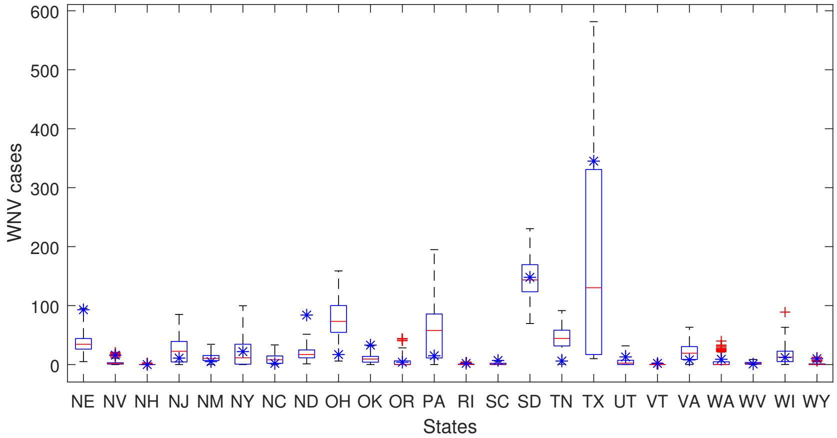

Fig D. WNV human incidence by states for 2016 from power-law influenced by flyway kernel model (for K=2.4233, $\beta_0 = 0.0029day^{-1}$, and $\lambda = 0.0452day^{-1}$), generated from 1000 simulation and observed data are indicated by blue colored star points. states name are given in short form. Simulated results are represented with a box plot in which the red horizontal lines show the median and the bottom and top edges of the boxes indicate 25th and 75th percentile respectively, The whiskers show the ranges of data points not considered outliers and outliers are showing by red + symbol.

Table B. Summary of evidence among three network models from ABC-SMC model selection algorithm for 2014, 2015 and 2016.

Evidence	2014	2015	2016
Power-law-flyway kernel network model against exponential kernel network model	positive	positive	positive
Power-law-flyway kernel network model against power-law kernel network model	positive	positive	strong

Discussion

The results from ABC-SMC (approximate Bayesian computation with sequential Monte Carlo sampling) model selection method are similar for 2014, 1015, and 2016. The results from ABC-SMC model selection method are summarized in Table SB. The ABC-SMC method selected power-law-flyway kernel as the best kernel than other two kernels. Power-law-flyway kernel can best describe the WNV human case data in the USA. The estimated parameters values from ABC-SMC parameter estimation method are slightly different for these three years. The reasons for this difference is the different initial condition, different host population, and seasonality (different temperature data).

References

1. Centers for Disease Control and Prevention;. Available from: https://www.cdc.gov/westnile/index.html.

2. Clements J, Schulenberg T, Iliff M, Roberson D, Fredericks T, Sullivan B, et al. The eBird/Clements checklist of birds of the world: v2015. URL: http://www birds cornell edu/clementschecklist/download/IOC. 2015;.

3. Kass RE, Raftery AE. Bayes factors. Journal of the american statistical association. 1995;90(430):773–795.
