# Supplementary material for: A spatio-temporal individual-based network framework for West Nile virus in the USA: Spreading pattern of West Nile virus
Source: PLoS Comput Biol. 2019 Mar 13;15(3):e1006875. doi: 10.1371/journal.pcbi.1006875 (PMC6433293; doi:10.1371/journal.pcbi.1006875)
Supplement: S2 Text — (PDF) [file pcbi.1006875.s002.pdf]

Approximate Bayesian Computation based on sequential Monte Carlo sampling (ABC-SMC) method

This research has used ABC-SMC methods to estimate parameters and to select best kernel network model among three distance dispersal kernels.

Parameter estimation

The steps for approximate Bayesian computation with sequential Monte Carlo sampling (ABC-SMC) algorithm for parameter estimation are [1–6]:

Step 1 Initialize tolerance ϵ for each SMC round, where $\epsilon_P < ..... < \epsilon_2 < \epsilon_1$. Set Population indicator, p=1.

Step 2 Particle indicator, n=1.

Step 3 Generate a particle (set of parameters),θ_p^n

(a). if p=1, sample from prior of parameters, $\pi(\theta)$;

(b). if p>1, sample the particle from previous population $\{\theta_{p-1}^n\}$ with weights W_{p-1} and then perturb the particle, $\theta^{'}$ by using perturbation kernel, PK_p to get $\theta^{''}$.

(c). if $\pi(\theta^{''}) == 0$, return to Step 3.

Step 4 Run the model R times with the new particle and compare the simulated weekly human WNV incidence with observed weekly WNV incidence using the goodness-of- fit metric, We calculated $r_p(\theta^{''}) = (1/R) * \sum_{r=1}^{R} 1(dist(x,d) < \epsilon_p)$, if $r_p(\theta^{''}) == 0$ reject the particle; go back to Step 3(a).

Step 5 Calculate the weight for the accepted particle,

(a). if p=1, $W_{n,p} = r_p(\theta^{''})$;

(b). if p>1, the weight is given by, $W_{n,p} = \frac{\pi(\theta_p^i) * r_p(\theta^{''})}{\sum_{j=1}^{N} W_{i,p-1} PK_p(\theta_{p-1}^j, \theta_p^i)}$.

Step 6 Repeat steps 3 - 5 until N= 1000 particles have been accepted.

Step 7 Normalize the weights. If $p < P$, set p= p+1, go to Step 2.

Model seclection

The steps for approximate Bayesian computation with sequential Monte Carlo sampling (ABC-SMC) algorithm for model selection are [1–6]:

Step 1 Initialize tolerance for each SMC round $\epsilon_P < ..... < \epsilon_2 < \epsilon_1$. Set Population indicator, p=1.

Step 2 Particle indicator, n=1.

Step 3 Generate a particle

(a). if p=1, sample model parameter m and parameters for that model from prior, $\pi(m, \theta)$;

(b). if p>1, sample model $m^{'}$ with probability $Pr_{t-1}(m^{'})$ and then perturb by perturbation kernel PKm_p, sample the particle from previous population $\{\theta(m^{''})_{p-1}\}$ with weights W_{p-1} and then perturb the particle $\theta^{'}$ by using perturbation kernel to get $\theta^{''}$.

(c). if $\pi(m^{''}, \theta^{''}) == 0$, return to Step 3.

Step 4 Run the model $m^{''}$, R times with the new particle and compare the simulated weekly human WNV incidence with observed weekly WNV incidence using the goodness-of- fit metric, We calculated $r_p(\theta^{''}) = (1/R) * \sum_{r=1}^{R} 1(dist(x, d) < \epsilon_p)$, if $r_p(\theta^{''}) == 0$ reject the particle; go back to Step 3.

Step 5 Calculate weight for the accepted particle, set $(m_p^n, \theta_p^n) = (m^{''}, \theta^{''})$,

(a). if p=1, $W_{n,p}(m_p^n, \theta_p^n) = (1/R) * \sum_{r=1}^{R} 1(dist(x, d) < \epsilon_p)$; Here R is the number of replicate simulation run for a fixed particle.

(b). if p>1, the weight is given by,

$$W_{n,p}(m_p^n, \theta_p^n) = \frac{\pi(m_p^n, \theta_p^n) * (1/R) * \sum_{r=1}^{R} 1(dist(x,d) < \epsilon_p)}{\sum_{j=1}^{N} W_{i,p-1} PK_p(\theta_{p-1}^j, \theta_p^i)}.$$

Step 6 Repeat steps 3 - 5 until N= 1000 particles have been accepted.

Step 7 Normalize the weights for every m. If $p < P$, set p= p+1, go to Step 3.

References

1. Toni T, Welch D, Strelkowa N, Ipsen A, Stumpf MP. Approximate Bayesian computation scheme for parameter inference and model selection in dynamical systems. Journal of the Royal Society Interface. 2009;6(31):187–202.

2. Csilléry K, Blum MG, Gaggiotti OE, François O. Approximate Bayesian computation (ABC) in practice. Trends in ecology & evolution. 2010;25(7):410–418.

3. Beaumont MA. Approximate Bayesian computation in evolution and ecology. Annual review of ecology, evolution, and systematics. 2010;41:379–406.

4. Sunnåker M, Busetto AG, Numminen E, Corander J, Foll M, Dessimoz C. Approximate bayesian computation. PLoS computational biology. 2013;9(1):e1002803.

5. Del Moral P, Doucet A, Jasra A. Sequential monte carlo samplers. Journal of the Royal Statistical Society: Series B (Statistical Methodology). 2006;68(3):411–436.

6. Sisson SA, Fan Y, Tanaka MM. Sequential monte carlo without likelihoods. Proceedings of the National Academy of Sciences. 2007;104(6):1760–1765.
