# Supplementary material for: A spatio-temporal individual-based network framework for West Nile virus in the USA: Spreading pattern of West Nile virus
Source: PLoS Comput Biol. 2019 Mar 13;15(3):e1006875. doi: 10.1371/journal.pcbi.1006875 (PMC6433293; doi:10.1371/journal.pcbi.1006875)
Supplement: S3 Text — (PDF) [file pcbi.1006875.s003.pdf]

Network Description

Table A. Description of sub-networks for 2014. $|V| = 9373$ and scaling constant $S_c = 0.03$ for 2014

Sub-network Id	Location	Nodes (% of $|V|$)	Probability of Active nodes				
			Jun.	Jul.	Aug.	Sept.	Oct.
SN1	AL	0.4588 %	0.4651	0.4884	0.4186	0.9767	1.0000
SN2	AZ	0.5334 %	1.0000	0.7600	0.4600	0.5200	0.5800
SN3	AR	0.3521 %	0.6970	1.0000	0.8182	0.6061	1.0000
SN4	CA	5.4732 %	0.6121	0.3996	0.3801	0.5166	1.0000
SN5	CO	2.2298 %	1.0000	1.0000	0.9139	1.0000	1.0000
SN6	CT	2.2511 %	1.0000	0.9716	0.7109	0.7630	1.0000
SN7	DE	1.4510 %	0.2868	0.3529	0.6397	1.0000	1.0000
SN8	DC	1.6964 %	0.2013	0.2830	0.6289	1.0000	0.8742
SN9	FL	0.1174 %	0.5455	0.4545	0.6364	0.6364	1.0000
SN10	GA	1.6003 %	0.5867	0.6600	0.5067	0.8400	1.0000
SN11	ID	0.6401 %	1.0000	1.0000	0.5333	1.0000	1.0000
SN12	IL	8.5778 %	0.7923	0.7923	0.8022	1.0000	1.0000
SN13	IN	1.7177 %	1.0000	0.9503	0.6584	0.9814	1.0000
SN14	IA	0.8855 %	0.8554	1.0000	0.7108	0.8313	1.0000
SN15	KS	1.2696 %	0.5378	0.7311	0.5882	1.0000	1.0000
SN16	KY	1.0135 %	0.9263	0.7158	0.8947	1.0000	1.0000
SN17	LA	0.2454 %	0.4783	0.4348	0.4348	0.5217	1.0000
SN18	ME	0.8322 %	1.0000	1.0000	0.5256	0.7308	1.0000
SN19	MD	3.3180 %	0.7878	0.8103	0.8842	1.0000	1.0000
SN20	MA	4.6623 %	0.8558	1.0000	0.9085	1.0000	1.0000
SN21	MI	4.0435 %	1.0000	0.9367	0.9604	1.0000	1.0000
SN22	MN	1.4403 %	1.0000	0.8296	0.6148	1.0000	1.0000
SN23	MS	0.1814 %	0.8235	0.5882	0.5882	0.6471	1.0000
SN24	MO	2.5605 %	0.5458	0.5417	0.4958	1.0000	1.0000
SN25	MT	1.3016 %	1.0000	1.0000	0.7131	0.6475	0.3689
SN26	NE	0.9175 %	1.0000	0.8837	0.6512	0.8488	1.0000
SN27	NV	0.3201 %	0.9667	0.7667	0.8000	1.0000	1.0000
SN28	NH	0.6828 %	1.0000	1.0000	0.6875	1.0000	1.0000
SN29	NJ	4.0435 %	0.6544	0.6781	0.7018	1.0000	1.0000
SN30	NM	0.5975 %	0.8036	0.6429	0.7857	1.0000	1.0000
SN31	NY	7.8843 %	0.8917	0.9107	1.0000	1.0000	1.0000
SN32	NC	1.4830 %	0.8633	0.7050	0.6835	1.0000	1.0000
SN33	ND	0.3627 %	1.0000	0.7647	1.0000	1.0000	1.0000
SN34	OH	4.4063 %	0.9637	1.0000	0.9274	1.0000	1.0000
SN35	OK	1.3016 %	0.2295	0.3033	0.3361	0.4098	1.0000
SN36	OR	2.0911 %	1.0000	1.0000	0.7041	1.0000	1.0000
SN37	PA	5.9853 %	0.8824	0.9037	1.0000	1.0000	1.0000
SN38	RI	0.4801 %	0.8000	1.0000	0.6444	0.8667	1.0000
SN39	SC	0.5334 %	0.4600	0.4600	0.5000	0.6600	1.0000
SN40	SD	0.4481 %	0.9762	1.0000	0.7619	0.9286	1.0000
SN41	TN	1.4403 %	0.6296	0.9259	0.8000	1.0000	1.0000

Table A. Description of sub-networks for 2014. $|V| = 9373$ and scaling constant $S_c = 0.03$ for 2014

Sub-network Id	Location	Nodes (% of $\|V\|$)	Probability of Active nodes				
SN42	TX	1.6003 %	0.2333	0.3800	0.5333	0.6533	1.0000
SN43	UT	1.2056 %	1.0000	0.7345	0.3894	0.5044	0.8142
SN44	VT	0.8322 %	1.0000	1.0000	0.7436	0.8462	1.0000
SN45	VA	5.3558 %	0.3665	0.3546	0.5936	1.0000	1.0000
SN46	WA	3.8622 %	1.0000	1.0000	0.5884	1.0000	1.0000
SN47	WV	0.5868 %	1.0000	0.8000	0.4727	0.5455	1.0000
SN48	WI	4.2462 %	1.0000	0.7236	0.5829	0.9020	1.0000
SN49	WY	0.4801 %	1.0000	1.0000	0.8000	0.7778	0.3111

Table B. Description of sub-networks for 2015. $|V| = 7657$ and scaling constant $S_c = 0.02$ for 2015

Sub-network Id	Location	Nodes (% of $\|V\|$)	Probability of Active nodes				
			Jun.	Jul.	Aug.	Sept.	Oct.
SN1	AL	0.2612%	1.000	0.9000	0.9000	0.9956	1.000
SN2	AZ	0.6138%	1.000	0.9149	0.6595	0.7021	0.5106
SN3	AR	0.4832%	0.9730	0.6757	1.000	0.7027	1.000
SN4	CA	3.1213%	1.000	0.6192	0.6443	0.6066	1.000
SN5	CO	3.3172%	1.000	0.6181	0.4645	0.9094	0.7834
SN6	CT	2.1027%	1.000	0.8695	0.6894	0.6894	1.000
SN7	DE	0.8620%	1	0.6969	0.9242	1.0000	1.000
SN8	DC	0.7183%	0.5636	0.5636	1.0000	0.9989	1.000
SN9	FL	0.1437%	0.9091	1.0000	1.0000	0.9911	1.000
SN10	GA	1.0317%	1.0000	0.8101	0.7848	0.8354	1.000
SN11	ID	1.2146%	1.0000	0.7204	0.6344	1.0000	0.6666
SN12	IL	6.6083%	1.0000	0.7608	0.8023	1.0000	1.000
SN13	IN	1.3974%	1.0000	0.9345	0.8317	1.0000	1.000
SN14	IA	0.8880%	1.0000	0.6764	0.7794	1.0000	1.000
SN15	KS	0.9011%	1.0000	0.5942	0.8260	1.0000	1.000
SN16	KY	1.0709%	0.7073	0.7682	1.0000	1.0000	1.000
SN17	LA	0.2742%	0.6190	0.7619	1.0000	0.9978	1.000
SN18	ME	1.0709%	1.0000	0.8414	0.5609	0.4878	1.000
SN19	MD	3.3433%	0.6914	0.5898	1.0000	0.8906	1.000
SN20	MA	3.9963%	0.7778	0.8725	1.0000	1.0000	1.000
SN21	MI	4.3620%	1.0000	0.8353	0.6946	1.0000	1.000
SN22	MN	1.3974%	1.0000	0.7196	0.9065	0.9111	1.000
SN23	MS	1.7239%	1.0000	0.8461	0.9230	0.9876	1.000
SN24	MO	1.7239%	0.9697	0.8181	1.0000	0.9976	1.000
SN25	MT	2.8862%	1.0000	0.4117	0.7013	0.7058	0.1719
SN26	NE	0.8358%	1.0000	0.8437	0.6718	1.0000	1.000
SN27	NV	0.3917%	1.0000	0.6333	0.6000	0.7333	1.0000
SN28	NH	0.6007%	1.0000	0.9130	0.6956	0.9876	1.0000
SN29	NJ	4.4142%	0.5769	0.5118	1.0000	0.9977	1.0000
SN30	NM	0.6007%	1.0000	0.6739	0.8043	0.9767	1.0000
SN31	NY	7.9404%	0.8125	0.7960	1.0000	0.9876	1.0000

Table B. Description of sub-networks for 2015. $|V| = 7657$ and scaling constant $S_c = 0.02$ for 2015

Sub-network Id	Location	Nodes (% of $\|V\|$)	Probability of Active nodes				
SN32	NC	1.3582%	1.0000	0.7788	0.7692	0.9879	1.0000
SN33	ND	0.7444%	1.0000	0.5087	0.9473	0.9871	0.6491
SN34	OH	4.3750%	1.000	0.8328	0.8746	0.9899	1.0000
SN35	OK	0.5093%	0.7949	1.0000	0.8461	0.8974	1.0000
SN36	OR	2.9776%	1.0000	0.6403	0.4210	0.9342	1.0000
SN37	PA	10.1475%	0.5534	0.9317	1.0000	0.7657	1.0000
SN38	RI	0.5876%	0.9556	1.0000	0.9333	0.9777	1.0000
SN39	SC	0.3395%	0.730	0.7692	1.0000	1.0000	1.0000
SN40	SD	0.6921%	1.0000	0.6226	0.3962	0.8113	1.0000
SN41	TN	1.0317%	0.9873	0.9240	1.0000	1.0000	1.0000
SN42	TX	1.1884%	0.3846	0.4835	1.0000	1.0000	1.0000
SN43	UT	1.319054%	1.0000	0.5049	0.3366	0.5148	0.5049
SN44	VT	1.0709%	1.0000	0.7804	0.5365	0.6219	1.0000
SN45	VA	2.6119%	0.7550	0.6600	1.0000	0.9950	1.0000
SN46	WA	4.2575%	1.0000	0.6809	0.5920	0.9190	1.0000
SN47	WV	0.5876%	1.0000	0.8666	0.6444	0.4444	1.0000
SN48	WI	6.6475%	1.0000	0.6620	0.4027	0.6149	0.6699
SN49	WY	0.8097%	1.0000	0.5967	0.8064	0.5483	0.2580

Table C. Description of sub-networks for 2016. $|V| = 7430$ and and scaling constant $S_c = 0.015$ for 2016

Sub-network Id	Location	Nodes (% of $\|V\|$)	Probability of Active nodes				
			Jun.	Jul.	Aug.	Sept.	Oct.
SN1	AL	0.6999 %	0.3654	0.2885	0.4423	0.5962	1.0000
SN2	AZ	0.5922 %	1.0000	0.6364	0.4545	0.3409	1.0000
SN3	AR	1.2113 %	0.2778	0.1556	0.1556	0.2111	1.0000
SN4	CA	2.7052 %	1.0000	0.6667	0.5423	0.5721	0.7363
SN5	CO	3.3513 %	1.0000	0.5783	0.4257	0.9036	1.0000
SN6	CT	1.8708 %	1.0000	0.6835	0.5108	0.6259	1.0000
SN7	DE	1.3324 %	0.2828	0.2828	0.3333	1.0000	1.0000
SN8	DC	0.6999 %	0.5000	0.4423	0.8077	1.0000	1.0000
SN9	FL	0.7402 %	0.0909	0.0909	0.0909	0.0909	1.0000
SN10	GA	1.5882 %	0.3898	0.3559	0.3305	0.4576	1.0000
SN11	ID	0.6864 %	1.0000	1.0000	0.4902	1.0000	1.0000
SN12	IL	8.1157 %	0.6683	0.5605	0.5423	1.0000	1.0000
SN13	IN	1.8708 %	0.8561	0.6547	0.5324	1.0000	1.0000
SN14	IA	0.8210 %	1.0000	0.8033	0.5738	0.9016	1.0000
SN15	KS	0.9421 %	0.7000	0.4429	0.4714	1.0000	1.0000
SN16	KY	1.1440 %	0.5647	0.5647	0.5765	1.0000	1.0000
SN17	LA	0.2153 %	0.5000	0.4375	0.5000	0.6250	1.0000
SN18	ME	0.7672 %	1.0000	1.0000	0.4561	0.6842	1.0000
SN19	MD	2.9206 %	0.7512	0.6083	0.6221	1.0000	1.0000
SN20	MA	4.8991 %	0.7555	0.6978	0.6951	1.0000	1.0000
SN21	MI	3.9435 %	1.0000	0.7782	0.8055	1.0000	1.0000

Table C. Description of sub-networks for 2016. $|V| = 7430$ and and scaling constant $S_c = 0.015$ for 2016

Sub-network Id	Location	Nodes (% of $\|V\|$)	Probability of Active nodes				
SN22	MN	1.8170 %	1.0000	0.5630	0.4444	1.0000	1.0000
SN23	MS	0.1750 %	0.4615	0.5385	0.5385	0.6923	1.0000
SN24	MO	2.1803 %	0.5741	0.4691	0.5185	1.0000	1.0000
SN25	MT	1.3190 %	1.0000	1.0000	0.8571	1.0000	0.6633
SN26	NE	0.8479 %	1.0000	0.4762	0.5079	0.8730	1.0000
SN27	NV	0.3634 %	1.0000	0.5926	0.3333	0.4074	0.8148
SN28	NH	0.5518 %	1.0000	1.0000	0.5854	1.0000	1.0000
SN29	NJ	3.9973 %	0.6801	0.4276	0.4377	1.0000	1.0000
SN30	NM	0.4980 %	1.0000	0.8108	0.6486	0.7838	1.0000
SN31	NY	6.6891 %	0.9819	0.8692	1.0000	1.0000	1.0000
SN32	NC	1.5612 %	0.5776	0.4741	0.4828	1.0000	1.0000
SN33	ND	0.6326 %	1.0000	0.5745	0.8723	1.0000	1.0000
SN34	OH	4.4145 %	0.9787	0.8780	0.7713	1.0000	1.0000
SN35	OK	1.3728 %	0.2647	0.2549	0.3039	0.4510	1.0000
SN36	OR	2.0054 %	1.0000	1.0000	0.5101	0.8322	1.0000
SN37	PA	8.7079 %	0.6955	0.5363	0.6584	1.0000	1.0000
SN38	RI	0.5787 %	1.0000	0.8372	0.4419	0.5581	0.9070
SN39	SC	0.5653 %	0.3333	0.2857	0.2619	0.4286	1.0000
SN40	SD	0.6191 %	1.0000	0.4348	0.3913	0.6304	1.0000
SN41	TN	3.3647 %	0.2680	0.2400	0.2920	0.4520	1.0000
SN42	TX	1.9246 %	0.1958	0.2587	0.3986	0.3846	1.0000
SN43	UT	1.0902 %	1.0000	0.6173	0.3704	0.5556	0.7654
SN44	VT	0.7268 %	1.0000	1.0000	0.6667	0.8148	1.0000
SN45	VA	2.7322 %	1.0000	0.6059	0.6158	0.8325	1.0000
SN46	WA	1.9112 %	1.0000	1.0000	1.0000	1.0000	1.0000
SN47	WV	0.5653 %	1.0000	0.7381	0.4762	0.6429	0.9762
SN48	WI	7.2275 %	1.0000	0.4991	0.2048	0.2365	0.5270
SN49	WY	0.4441 %	1.0000	1.0000	0.8788	1.0000	0.4545
