# Supplementary material for: A spatio-temporal individual-based network framework for West Nile virus in the USA: Spreading pattern of West Nile virus
Source: PLoS Comput Biol. 2019 Mar 13;15(3):e1006875. doi: 10.1371/journal.pcbi.1006875 (PMC6433293; doi:10.1371/journal.pcbi.1006875)
Supplement: S4 Text — (PDF) [file pcbi.1006875.s004.pdf]

Architecture of the framework

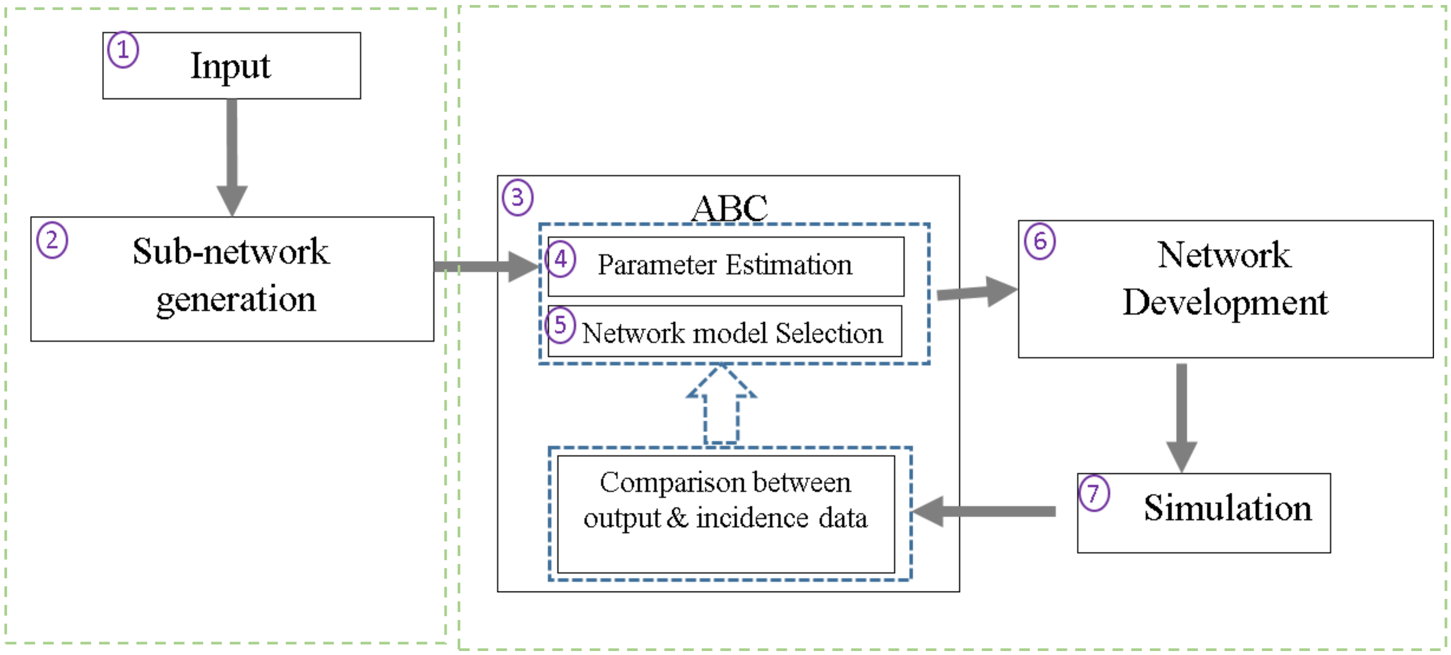

1 Input: a. Geographic locations of the contiguous United States plus the District of Columbia. (source: https://developers.google.com/public-data/docs/canonical/states_csv)

b. Population of the American Robin in each states. (source: https://ebird.org/GuideMe?bMonth=1&eMonth=12&bYear=2015&eYear=2015&speciesCodes=amerob&getLocations=states&states=US-CA&reportType=species&changeLocation.x=89&changeLocation.y=2)

c. Average monthly temperature by states. (source: https://www.ncdc.noaa.gov/cag/)

d. Weekly human incidence data for each states for WNV. (source: https://data.cdc.gov/NNDSS/NNDSS-Table-II-West-Nile-virus-disease/r7hc-32zu/data.)

2 Sub-Network Generation: Sub-network creation for each state based on the American Robin population data (Methods in the Network framework section).

3 ABC: Approximate Bayesian Computation was used for the parameter estimation and to select a network kernel model. Algorithm for approximate Bayesian computation is provided in the Text S2.

6 Network Development: Connections among and between sub-networks were formed according to the selected network kernel model (a. exponential kernel network model, b. power-law kernel network model, and c. power-law biased by flyway network model).

7 Simulation: The selected network kernel model was simulated with the estimated parameters. We used the Generalized Epidemic Mean-Field (GEMF) model developed by Network Science and Engineering group for simulations. The code is available in https://www.ece.k-state.edu/netse/software/index.html. The output of this block is the weekly WNV human incidence, which has used in ABC block to compare the simulated result with the actual observed human incidence data.
